# The Effect of Post-mastectomy Radiotherapy in Patients With Metaplastic Breast Cancer: An Analysis of SEER Database

**DOI:** 10.3389/fonc.2019.00747

**Published:** 2019-08-12

**Authors:** Jun Wang, Wen-Wen Zhang, Chen-Lu Lian, Jia-Yuan Sun, Zhen-Yu He, San-Gang Wu

**Affiliations:** ^1^Department of Radiation Oncology, Cancer Hospital, The First Affiliated Hospital of Xiamen University, Teaching Hospital of Fujian Medical University, Xiamen, China; ^2^State Key Laboratory of Oncology in South China, Department of Radiation Oncology, Collaborative Innovation Center of Cancer Medicine, Sun Yat-sen University Cancer Center, Guangzhou, China

**Keywords:** breast neoplasms, radiotherapy, mastectomy, lymph node metastasis, survival, SEER

## Abstract

**Introduction:** Metaplastic breast cancer (MBC) is a rare and aggressive form of breast cancer. The present study aimed to assess the effect of post-mastectomy radiotherapy (PMRT) in MBC patients with intermediate-risk (T1-2N1M0 and T3N0M0) and high-risk (T1-4N2-3M0 and T4N0-1M0) disease.

**Methods:** The Surveillance, Epidemiology and End Results database was used to analyze patients with MBC between 2000 and 2014. Kaplan–Meier analysis, log-rank tests, and the multivariate Cox proportional model were used for statistical analysis.

**Results:** We identified 460 patients with a median follow-up time of 31 months (range, 2–178 months). Five-year breast cancer specific survival (BCSS) for all patients was 57.5%. In the entire group, multivariate analysis showed that PMRT was associated with better BCSS (hazard ratio (HR) 0.500, 95% confidence interval (CI) 0.366–0.683, *P* < 0.001). The 5-year BCSS in PMRT and non-PMRT groups were 62.3 and 50.3%, respectively (*P* = 0.001). When stratified the patients into intermediate-risk and high-risk groups, PMRT could improve BCSS compared with that in non-PMRT patients in both the intermediate- and high-risk groups. For the intermediate-risk group, the 5-year BCSS was 74.3 and 64.7% in PMRT and non-PMRT groups (*P* = 0.042), respectively, and was 52.1 and 28.8% in high-risk patients treated with PMRT and non-PMRT, respectively (*P* < 0.001).

**Conclusion:** PMRT could improve the BCSS of MBC patients with intermediate- and high-risk disease.

## Background

Metaplastic breast cancer (MBC) was identified as a unique pathological type of breast cancer by the World Health Organization in 2000, and the rate of MBC diagnosis has increased ever since. MBC is a rare disease with aggressive biological behavior, accounting for 0.25–1% of all breast cancers (1, 2), and is characterized by either a homogeneous population or mixtures of squamous cell carcinoma, adenocarcinoma, epithelial, and mesenchymal components (3–8). In addition, patients with MBC have distinct histopathological and molecular signatures, including larger tumor size; less frequently with axillary nodal metastases; triple-negative disease; and higher Ki-67, p53, CK5/6, and EGFR expression levels (9–11). Several previous studies have indicated that the survival of patients with MBC was significantly lower than in those with invasive ductal carcinomas (IDC) (11–14).

The optimal management of MBC remains controversial due to the rarity of this disease. In the current practice, most patients were treated with mastectomy because of the larger tumor size associated with this disease (15). The role of post-mastectomy radiotherapy (PMRT) might be important in this patient subset because of about 28–46% of patients may develop locoregional recurrence (LRR) after surgery (10, 12, 16). However, the effect of PMRT in MBC is a matter of debate. Several studies showed better outcomes in PMRT groups (15, 17–20), while other studies indicated that the receipt of PMRT was not associated with better outcomes (2, 21, 22). The main reasons for above conflicting results might be the difference in sample sizes and treatment patterns of the study populations. In addition, the recommendation to use of PMRT is also controversial in patients with intermediate-risk disease (T1-2N1M0 and T3N0M0) (2). Consequently, the present study was aimed to assess the role of PMRT in MBC, especially in patients with intermediate-risk, using a real-world population-based database (Surveillance, Epidemiology, and End Results, SEER).

## Materials and Methods

### SEER Database and Patients

Data were obtained from the SEER database of the National Cancer Institute, which is an open access resource with 18 population-based cancer registries of patients in the United States for cancer-based epidemiology and survival. Women with intermediate-risk and high-risk MBC (23) treated with mastectomy and chemotherapy from 2000 to 2014 were identified. The code for MBC is 8,575 in the SEER database, according to the third edition of International Classification of Diseases for Oncology. The intermediate-risk group included patients with stage T1-2N1M0 and T3N0M0 disease (23), and the high-risk group included patients with stage T1-4N2-3M0 and T4N0-1M0 disease (24). Patients with available race/ethnicity, tumor (T) stage, nodal (N) stage, and having records on whether they received PMRT were included. We excluded patients without a positive histology diagnosis and receipt of non-beam irradiation. Using the SEER data is exempt from the approval process of Institutional Review Board.

### Variables

We included the following patient demographic and clinicopathological variables: age ( ≤ 50 years, > 50 years), race/ethnicity (Non-Hispanic white, Non-Hispanic Black, Hispanic, and Other), T stage (T1, T2, T3, and T4), N stage (N0, N1, N2, and N3), risk stratification (intermediate-risk, high-risk), estrogen receptor (ER) status, progesterone receptor (PR) status, and human epidermal growth factor receptor-2 (HER2) status. The definition of TNM (T-tumor, N-node, M-metastasis) stage was according to the six edition of the Union for International Cancer Control /American Joint Committee on Cancer pathologic staging system. We only included the status of HER-2 after 2010 because SEER only recorded these data after 2010. The primary end point of this study was breast cancer specific survival (BCSS), which was calculated as the date from the diagnosis of MBC to death from breast cancer.

### Statistical Analysis

The χ2 test was used to analyze the differences between PMRT and non-PMRT groups. Kaplan–Meier analysis and log-rank testing were used to compare BCSS curves. The risk factors for BCSS were assessed using the Cox proportional hazards model. All calculations were performed using SPSS statistical software (version 22.0; IBM Corporation, Armonk, NY, USA), and *P* < 0.05 was considered statistically significant.

## Results

### Patient Characteristics

A total of 460 patients were identified. The characteristics of the patients in the study population are presented in Table 1. The surgical procedures of the 460 patients are listed in Table 2. Among all the patients, 73% of them were aged <50 years, with a median age of 57 years (range, 27–88 years). Most of them were Non-Hispanic White (*n* = 265, 57.6%), poor differentiation/undifferentiated (*n* = 374, 81.3%), ER negative (*n* = 358, 78.8%), PR negative (*n* = 389, 84.6%), and HER2 negative (*n* = 196, 92% in HER2 available patients). In patients with available breast cancer subtype information, 65.3% were triple-negative patients. A total of 20 (4.3%), 131 (28.5%), 201 (43.7%), and 108 (23.5%) of the patients had T1, T2, T3, and T4 stage disease, respectively. In addition, 171 (37.2%), 199 (43.3%), 59 (12.8%), and 31 (6.7%) patients had N0, N1, N2, and N3 stage disease, respectively.

**Table 1:** Patient demographic and clinical characteristics.

**Variables**	***N* (%)**	**Non-PMRT (%)**	**PMRT (%)**	***P***
**Age (years)**				
≤50	336 (73.0)	129 (69.4)	207 (75.5)	0.142
>50	124 (27.0)	57 (30.6)	67 (24.5)	
**Race/ethnicity**				
Non-hispanic white	265 (57.6)	113 (60.8)	152 (55.5)	0.487
Non-hispanic black	93 (20.2)	34 (18.3)	59 (21.5)	
Hispanic	64 (13.9)	27 (14.5)	37 (13.5)	
Other	38 (8.3)	12 (6.5)	26 (9.5)	
**Grade**				
Well/moderately differentiated	28 (6.1)	7 (3.8)	21 (7.7)	0.159
Poorly differentiated/undifferentiated	374 (81.3)	152 (81.7)	222 (81.0)	
Unknown	58 (12.6)	27 (14.5)	31 (11.3)	
**Tumor stage**				
T1	20 (4.3)	11 (5.9)	9 (3.3)	0.002
T2	131 (28.5)	69 (37.1)	62 (22.6)	
T3	201 (43.7)	68 (36.6)	133 (48.5)	
T4	108 (23.5)	38 (20.4)	70 (25.5)	
**Nodal stage**				
N0	171 (37.2)	60 (32.3)	111 (40.5)	0.129
N1	199 (43.3)	92 (49.5)	107 (39.1)	
N2	59 (12.8)	24 (12.9)	35 (12.8)	
N3	31 (6.7)	10 (5.4)	21 (7.7)	
**Risk stratification**				
Intermediate risk	233 (50.7)	111 (59.7)	122 (44.5)	0.001
High risk	227 (49.3)	75 (41.3)	152 (55.5)	
**ER status**				
Negative	358 (78.8)	142 (76.3)	216 (78.8)	0.662
Positive	84 (18.3)	35 (18.8)	49 (17.9)	
Unknown	18 (3.9)	9 (4.8)	9 (3.3)	
**PR status**				
Negative	389 (84.6)	156 (83.9)	233 (85)	0.904
Positive	51 (11.1)	21 (11.3)	30 (10.9)	
Unknown	20 (4.3)	9 (4.8)	11 (4.0)	
**HER-2 status (*****n*** **=** **213)**				
Negative	196 (92)	73 (93.6)	123 (91.1)	0.348
Positive	17 (8)	5 (6.4)	12 (8.9)	

*ER, estrogen receptor; HER-2, human epidermal growth factor receptor 2; PMRT, post-mastectomy radiotherapy; PR, progesterone receptor*.

**Table 2:** The surgical procedures of the 460 patients.

**Surgical procedures**	***n* (%)**
Subcutaneous mastectomy	1 (0.2)
Total (simple) mastectomy, not otherwise specified	105 (22.8)
Modified radical mastectomy	344 (74.8)
Radical mastectomy, not otherwise specified	7 (1.5)
Extended radical mastectomy	2 (0.4)
Mastectomy, not otherwise specified	1 (0.2)

Among the 460 patients, 59.6% (*n* = 274) of them received PMRT, and patients with advanced T stage (*P* = 0.002) and high-risk group (*P* = 0.001) were more likely to receive PMRT.

### Survival

**Figure 1:**
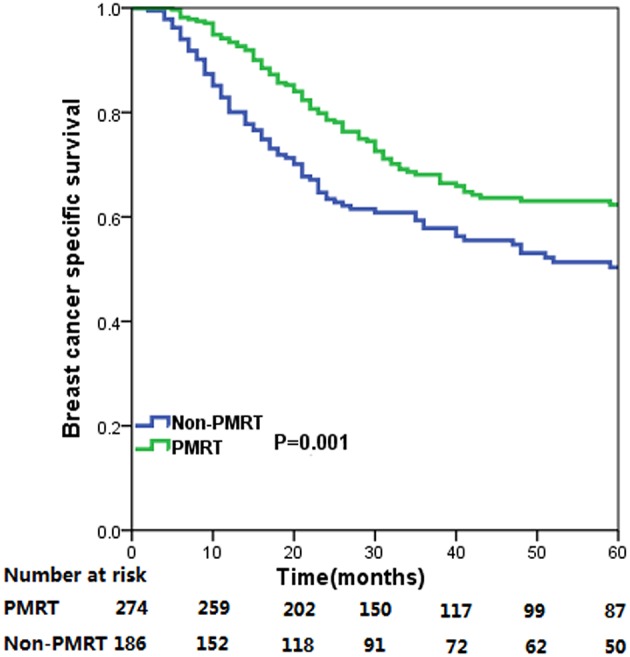
Breast cancer specific survival curves for all cases of metaplastic breast carcinoma with and without post-mastectomy radiotherapy.

**Figure 2:**
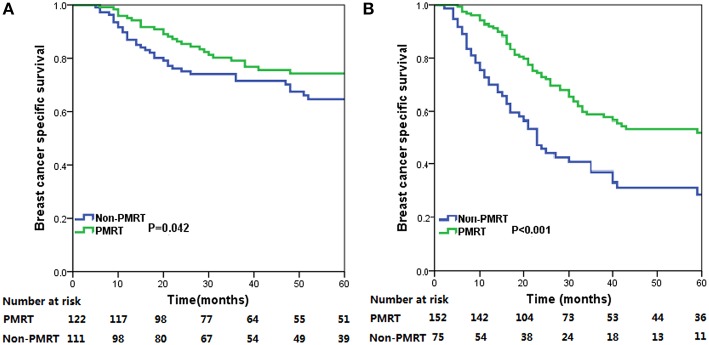
Breast cancer specific survival (BCSS) curves for all patients with metaplastic breast cancer (MBC) with and without post-mastectomy radiotherapy (PMRT) in the intermediate-risk **(A)** and high-risk **(B)** groups.

The median follow-up time for the entire cohort was 31 months (range, 2–178 months), and were 33.0 months (range, 5–178 months) and 29.5 months (range, 2–176 months) in PMRT group and non-PMRT group, respectively. A total of 250 deaths were observed, including 174 breast cancer related-deaths. The 5-year BCSS for all patients was 57.5%. The 5-year BCSS in the PMRT and non-PMRT groups were 62.3 and 50.3%, respectively (*P* = 0.001) (Figure 1). When stratified the patients into intermediate-risk and high-risk groups, PMRT could improve BCSS compared with that for patients in the non-PMRT group, in both the intermediate- and high-risk groups. For the intermediate-risk group, the 5-year BCSS were 74.3 and 64.7% in the PMRT and non-PMRT groups (*P* = 0.042) (Figure 2A), respectively, and were 52.1 and 28.8% in high-risk patients treated with PMRT and non-PMRT (*P* < 0.001), respectively (Figure 2B).

### Analysis of Prognostic Factors

We further analyzed the independent prognostic factors related to BCSS for MBC using the Cox proportional hazards model. In the entire group, multivariate analysis showed that PMRT was an independent prognostic factor related to better BCSS [hazard ratio [HR] 0.500, 95% confidence interval [CI] 0.366–0.683, *P* < 0.001] (Table 3). In addition, tumor stage and nodal stage were also independent indicators for BCSS. However, age, race/ethnicity, grade, ER status, and PR status were not related to BCSS.

**Table 3:** Multivariate analysis of prognostic factors in breast cancer specific survival.

**Variables**	**HR**	**95% CI**	***P***
**Age (years)**			
≤50	1		
>50	1.151	0.810–1.635	0.434
**Race/ethnicity**			
Non-hispanic white	1		
Non-hispanic black	0.763	0.511–1.141	0.188
Hispanic	0.690	0.428–1.112	0.128
Other	0.761	0.415–1.397	0.379
**Grade**			
Well/moderately differentiated	1		
Poorly differentiated/Undifferentiated	1.381	0.670–2.848	0.382
Unknown	1.508	0.662–3.435	0.329
**Tumor stage**			
T1	1		
T2	1.113	0.391–1.171	0.841
T3	2.943	1.047–8.269	0.041
T4	3.447	1.219–9.744	0.020
**Nodal stage**			
N0	1		
N1	1.365	0.911–2.046	0.131
N2	1.822	1.112–2.986	0.017
N3	2.157	1.160–4.009	0.015
**ER status**			
Negative	1		
Positive	0.715	0.433–1.180	0.190
**PR status**			
Negative	1		
Positive	1.266	0.704–2.277	0.431
**PMRT**			
No	1		
Yes	0.500	0.366–0.683	<0.001

*CI, confidence interval; ER, estrogen receptor; HR, hazard ratio, PMRT, post-mastectomy radiotherapy; PR, progesterone receptor*.

When stratified by the risk groups, PMRT was also an independent prognostic factor for BCSS in both the intermediate- and high-risk groups. Patients who received PMRT were associated with better BCSS compared with those in non-PMRT group in the intermediate-risk (HR = 0.466, 95% CI 0.280–0.774, *P* = 0.003) and high-risk (HR = 0.450 95% CI 0.301–0.675, *P* < 0.001) groups.

## Discussion

In the present study, we assessed the effect of PMRT in MBC, and our results found that receipt of PMRT was associated better BCSS in patients with intermediate-risk (T1-2N1M0 and T3N0M0) and high-risk (T1-4N2-3M0 and T4N0-1M0) disease.

We recognized homogeneity and heterogeneity in our study population. The results of our study were consistent with previous studies, which indicated that most patients with MBC patients presented with larger tumors, triple-negative disease, and higher tumor grade (9, 11, 16). In addition, we only included patients with intermediate- and high-risk cohorts, 19.5% of patients had stage N2 and N3 disease, and 80.5% of patients had N0 and N1 disease. Moreover, Pizza et al. reported that N0 stage was 78.1 vs. 65.7%, and N1 or above was 21.9 vs. 34.3% in MBC and IDC patients, respectively (*P* < 0.001) (1), which indicated that a high nodal involvement burden was less common in patients with MBC. Similar to previous study (25), we found that the advanced nodal stage was related to poor outcome in MBC, which was similar to the results for IDC (26).

The recurrence pattern of MBC has not been well-delineated in this rare disease. We were unable to assess the patterns of disease recurrence because of the lack of recurrence data in the SEER database. A previous study from the MD Anderson Cancer Center that included 47 patients with MBC showed that 28% of the patients developed LRR, with a median follow-up time of 30 months. However, the study included a limited number of patients, and this cohort may have received insufficient treatment (12). Another study from Korea that included 35 patients also showed that MBC had a higher recurrence rate of 46.8% compared with 9.3% in IDC patients, and MBC was a poor prognostic factor for disease recurrence (HR 3.89, 95% CI 1.36–11.14, *P* = 0.01) (10). Moreover, several previous studies found that the MBC subtype was more resistant to chemotherapy (27–29). Therefore, PMRT may play an important role in the management of MBC.

In the National Comprehensive Cancer Network breast cancer guidelines, PMRT is strongly recommended in T1-2N1 stage disease and is routinely used in patients with N2 stage disease (30). However, the guidelines were not stratified by histological subtypes. The percentage of PMRT receipt was only 58.1% in our study, and the percentage of PMRT receipt was markedly different in previous studies, ranging from 38.6 to 72% (2, 15, 17–20, 31), which indicated that the role of PMRT in patients with MBC is unclear, despite high recurrence.

The effect of PMRT in MBC remains controversial. Although some investigators have reported favorable prognosis in the PMRT group (15, 17–20), others have observed that adjuvant PMRT had no association with survival (21, 22). Haque et al. examined the effect of PMRT with MBC using the National Cancer Database and showed that 45.2% of the patients received PMRT, and that PMRT could lead to higher overall survival (OS) in patients with pT3–4/N+ disease (*p* < 0.001), but not in patients with pT1–2N0 disease (17).However, their observed end point was OS, which was affected by variety of uncontrollable factors and could not accurately reflect death from breast cancer. In addition, in a study from Tseng et al., which included 1,501 patients with stage I-IV MBC, the results of univariate analysis showed that post-operative radiotherapy was associated with better BCSS (*P* < 0.01) and OS (*P* = 0.003) in patients who received breast conserving surgery, whereas it was not related to better outcomes in patients who received PMRT. However, the results of multivariate analysis indicated that post-operative radiotherapy provided an OS benefit but not a BCSS benefit to patients receiving breast conserving surgery and mastectomy (2). The study by Tseng et al. also included patients with stage T1-2N0 and stage IV patients, which are not indications for PMRT in current clinical practice. Moreover, the information on chemotherapy receipt was not recorded in this study. Our study included patients with node-positive disease or larger tumor size (>5 cm) who received chemotherapy, and our findings suggested that PMRT could significantly improve BCSS in this patient subset.

The value of PMRT in patients with intermediate-risk invasive breast cancer has been controversial. A meta-analysis from the Early Breast Cancer Trialists Collaborative Group further supported the view that PMRT could reduce LRR and improve BCSS in patients with 1–3 positive lymph nodes (32). However, a recent study showed that survival in patients not receiving PMRT was comparable to that in patients receiving PMRT in the era of modern taxane-based chemotherapy (33). Whether the current chemotherapy practice could further determine the survival benefit of PMRT in patients with MBC remains controversial. We further analyzed whether the patients with MBC in the intermediate-risk group could benefit from PMRT, and found that PMRT was also associated with better BCSS in this population. However, because of the low incidence of MBC, it is difficult to conduct a large-scale randomized controlled trial. SUPREMO, the largest prospective trial to assess the value of PMRT in intermediate-risk groups, is ongoing, and MBC is not listed as an exclusion criterion. We anticipate the results of the stratified analysis to assess the value of PMRT in patients with intermediate-risk MBC in the era of systemic therapy.

There were several limitations in our study. First, it was a retrospective study with the observational nature and the possibility of selection bias, and patients were not randomly assigned to PMRT group and non-PMRT group. Second, the number of cycles and specific agents of chemotherapy, the sequence of chemotherapy and surgery, endocrine therapy, technique, dose, and target volume of PMRT as well as compliance to therapy were not included in the SEER database. Third, the patient baseline characteristics including performance status, comorbidities, and socioeconomic environments were also lacking in the SEER program. In addition, with a higher risk of death in MBC, the median follow-up time of our study was only 31 months, longer-term and prospective results are needed to draw definitive conclusions on the utilization of PMRT in MBC. However, the low incidence of MBC limits the possibility to conduct prospective clinical trials with large cohorts. Moreover, the patterns of LRR and distant recurrence were also missing from the SEER database. Finally, the percentage of PMRT receipt was under-reported in the SEER database (34). Therefore, our study may not be better than single institution retrospective data. However, the results of our study will contribute to the current knowledge of the role of PMRT in MBC.

## Conclusion

In conclusion, our study suggests that PMRT could improve BCSS in MBC patients with the intermediate- and high-risk groups. More prospective studies are required to confirm our results and to identify the optimal protocols for the use of PMRT in the management of MBC.

## Data Availability

Publicly available datasets were analyzed in this study. This data can be found here: www.seer.cancer.gov.

## Ethics Statement

This study was exempt from the approval processes of the Institutional Review Boards because the SEER database patient information is de-identified.

## Author Contributions

JW, Z-YH, S-GW, and W-WZ are lead authors who participated in data collection, manuscript drafting, table/figure creation, and manuscript revision. C-LL and J-YS are senior authors who aided in drafting the manuscript and manuscript revision. Z-YH and S-GW are the corresponding authors who initially developed the concept and drafted and revised the manuscript. All authors read and approved the final manuscript.

### Conflict of Interest Statement

The authors declare that the research was conducted in the absence of any commercial or financial relationships that could be construed as a potential conflict of interest.
